# mAIcrobe: an open-source framework for high-throughput bacterial image analysis

**DOI:** 10.1038/s44488-026-00022-y

**Published:** 2026-09-15

**Authors:** António D. Brito, Dominik Alwardt, Beatriz de P. Mariz, Sérgio R. Filipe, Mariana G. Pinho, Bruno M. Saraiva, Ricardo Henriques

**Affiliations:** 1 Instituto de Tecnologia Química e Biológica António Xavier, Universidade Nova de Lisboa, Oeiras, Portugal; 2 Faculdade de Ciências e Tecnologia, Universidade Nova de Lisboa, Lisbon, Portugal

**Keywords:** Computational biology and bioinformatics, Microbiology

## Abstract

Microscopy of bacterial cells is crucial for studying bacterial growth, cell division, or responses to antibiotics. However, analyzing these images can be challenging because bacteria vary widely in shape and behaviour. Many existing tools require specialised computational expertise, which can limit their accessibility. Here we present mAIcrobe, an open-source image analysis framework that makes advanced bacterial microscopy analysis more accessible by combining deep learning-based segmentation methods, including StarDist, CellPose, and U-Net, with quantitative morphological profiling and a flexible neural network-based classification model. mAIcrobe can analyse a wide range of bacterial species, from spherical *Staphylococcus aureus* to rod-shaped *Escherichia coli*, across different microscopy modalities, within a single environment. We demonstrate the utility of mAIcrobe by using it to identify antibiotic-induced changes in *E. coli* and cell cycle defects in *S. aureus* DnaA mutants. The framework is designed to be modular and extensible, with Jupyter notebooks provided to facilitate the development of custom models, thereby making AI-driven image analysis more accessible to the microbiology community.

## Introduction

Microscopy remains fundamental to microbial cell biology. However, quantitative analysis of bacterial images presents significant challenges. These include population heterogeneity, the small size of bacterial cells, morphological diversity among species, and the range of imaging techniques employed. Manual analysis constitutes a major bottleneck, as it is time-consuming, subjective and susceptible to human error, thereby limiting research throughput and reproducibility.

To overcome these limitations, we developed mAIcrobe, a comprehensive framework for bacterial image analysis. It supports multiple bacterial species, various microscopy modalities, and flexible, customisable analysis workflows. By integrating various segmentation methods, quantitative morphological measurements, and an adaptable classification model, mAIcrobe provides a powerful tool for a broad range of studies in bacterial cell biology. We have made our work accessible through the napari-mAIcrobe plugin, which is accompanied by Jupyter notebooks (Supplementary Table 1) to facilitate the training of custom classification models and step-by-step video tutorials to guide new users (Supplementary Table 2).

The field has seen several automated image analysis tools suited to bacterial images, from ImageJ^1^ plugins like MicrobeJ^2^ to standalone software such as Oufti^3^, OmniSegger^4^ and CellProfiler^5^, as well as more recent tools, such as micromorph^6^, a Python package and napari^7^ plugin. Our own contribution, eHooke^8^, provided an open-source solution for the semi-automated analysis of cocci, particularly *Staphylococcus aureus*. Although eHooke is a valuable tool for studying the cell cycle in spherical bacteria, its architecture constrained its applicability to other morphologies and made integration with new deep learning models challenging. DeepBacs^9^ has also made available several state-of-the-art artificial neural-network models tailored for bacterial microscopy using the ZeroCostDL4Mic platform^10^. The rapid evolution of bioimage analysis, coupled with the broad adoption of the napari ecosystem^7^, presented a clear opportunity to engineer a more powerful and extensible framework built on the modern scientific Python stack.

**Fig. 1:**
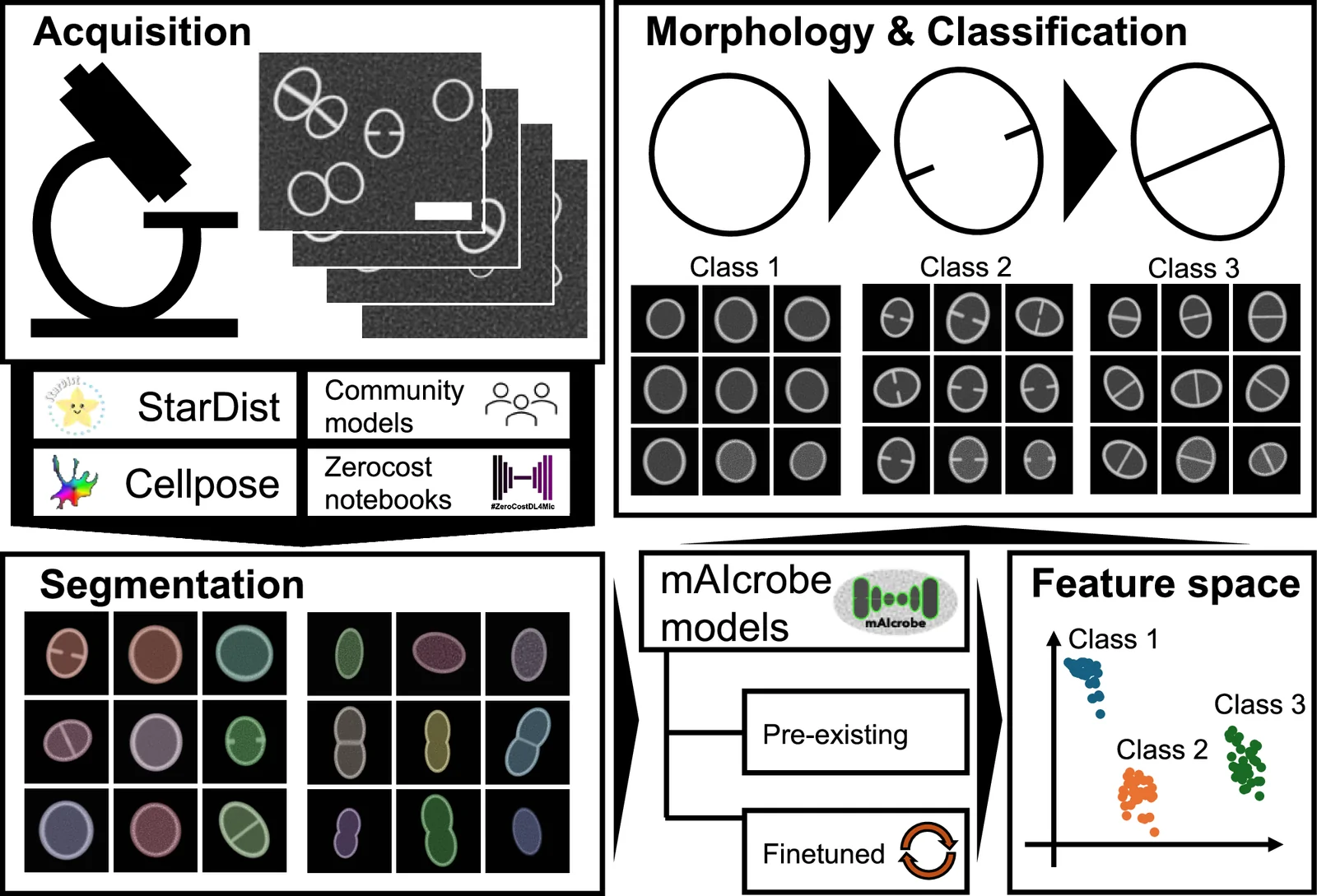
mAIcrobe workflow. After image acquisition, the napari-mAIcrobe plugin facilitates analysis through a user-friendly interface for segmentation, morphological measurements and classification using a variety of pre-trained or custom models.

We seized this opportunity to design mAIcrobe (Fig. 1), a next-generation platform prioritising versatility and performance. The design philosophy focused on overcoming morphological constraints, enabling the selection of optimal segmentation algorithms, and facilitating the rapid adaptation of deep learning models to address emerging biological questions. The napari framework was selected as the foundation for mAIcrobe due to its modular architecture and interactive visualisation capabilities, which align with these objectives.

## Results

Developed within the napari plugin ecosystem, mAIcrobe provides an intuitive and extensible platform for bacterial image analysis. The framework integrates image segmentation, morphological measurement and classification into a unified workflow. Its modular design enables the selection of segmentation models and classification strategies tailored to specific experimental requirements (Supplementary Table 3). A central feature of the napari-mAIcrobe plugin is its support for real-time visualisation and dynamic parameter adjustment, facilitating optimisation of image processing across diverse bacterial species and microscopy setups (Supplementary Fig. 1). The following sections illustrate these capabilities through selected biological applications.

### Segmentation

**Fig. 2:**
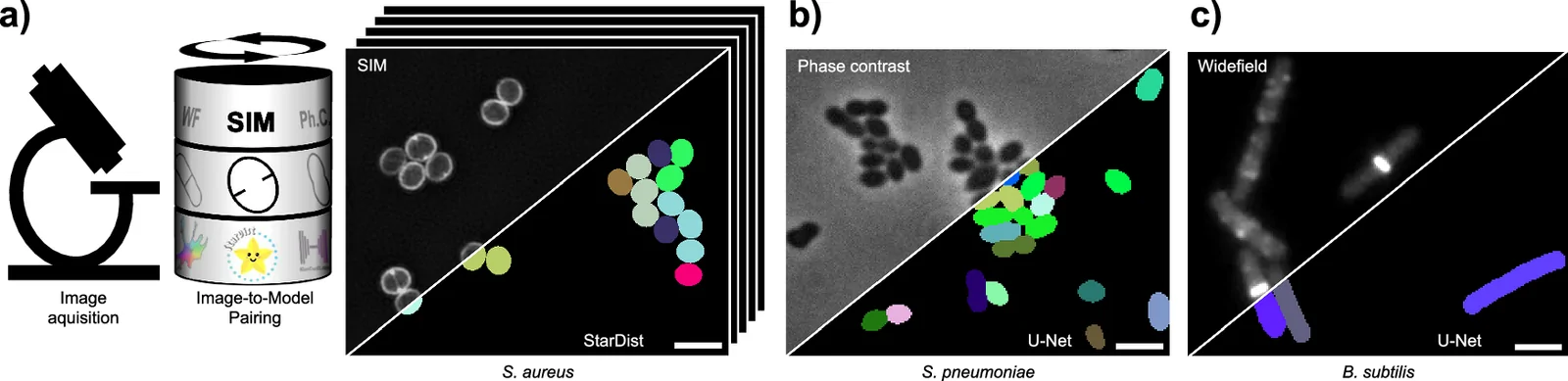
mAIcrobe segmentation capabilities. The platform can perform segmentation of images from a variety of bacterial species obtained using different microscopy modalities, using various segmentation models. **a** SIM image of *Staphylococcus aureus* JE2 strain labeled with membrane dye NileRed, with cells segmented using a StarDist model. **b** Phase-contrast image of *Streptococcus pneumoniae* cells segmented with a U-Net trained via ZeroCostDL4Mic. **c** Conventional fluorescence widefield microscopy image of a *Bacillus subtilis* strain expressing FtsZ-GFP, also segmented using a U-Net trained via ZeroCostDL4Mic. All scale bars are 2 µm.

mAIcrobe features a flexible segmentation engine designed to accommodate diverse bacterial species and microscopy modalities (Fig. 2 and Supplementary Table 4). The framework integrates several leading segmentation approaches, including StarDist^11^, CellPose^12^ and custom U-Net^13^ models trained using the ZeroCostDL4Mic framework^10^. This multi-model strategy enables the selection of the most suitable algorithm for specific experimental conditions and bacterial morphologies, thereby ensuring high-quality segmentation. Unlike tools limited to particular morphologies, mAIcrobe supports the analysis of rod-shaped, spherical and other bacterial forms within a single framework.

This versatility is demonstrated in several applications. In structured illumination microscopy (SIM) images of *S. aureus* labelled with membrane dye NileRed, the StarDist model achieves accurate cell boundary detection (Fig. 2a). For phase-contrast microscopy of *Streptococcus pneumoniae*, U-Net models trained with ZeroCostDL4Mic provide reliable segmentation of cells (Fig. 2b). The framework also processes conventional widefield fluorescence images, segmenting *Bacillus subtilis* expressing FtsZ-GFP (Fig. 2c)^9^. By integrating these models within a single interface, mAIcrobe eliminates the need to switch between software packages, thereby streamlining the identification of optimal segmentation approaches.

Importantly, reliable quantification always depends on adequate image quality, and segmentation performance should be validated on images of comparable quality to the experimental data. Nevertheless, the segmentation results obtained with mAIcrobe are robust to moderate image degradation, as accurate segmentation of *S. aureus* cells are obtained even when signal-to-noise ratio and resolution are reduced to half (Supplementary Fig. 2).

### Morphological measurements

Beyond segmentation, mAIcrobe performs quantitative morphological analysis of bacterial cell properties across diverse experimental conditions (Supplementary Table 3). From segmented cells, the framework extracts key morphological parameters, including cell area, perimeter and eccentricity, alongside multi-channel fluorescence intensity measurements. This quantitative data provides a solid basis for characterising cellular responses to drug treatments or genetic modifications. To ensure interoperability and support reproducible research, all results can be readily exported to standard formats, such as CSV, for downstream statistical analysis and visualisation.

**Fig. 3:**
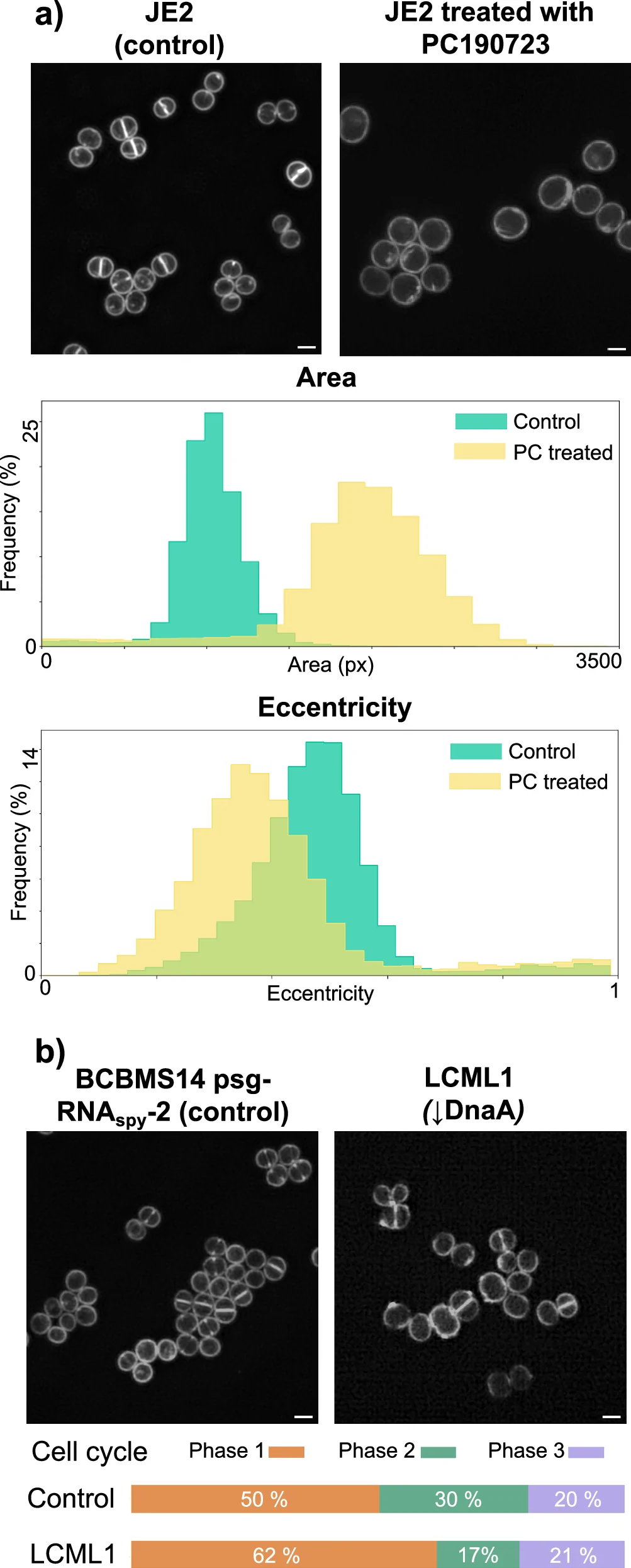
Quantitative phenotyping with mAIcrobe. mAIcrobe is capable of identifying phenotypic variations in microbial cells. **a** Morphological changes of *S. aureus* cells treated with the antibiotic PC190723, which leads to larger and rounder cells. The histograms show cell area and eccentricity for control (green, *n* = 12,831 cells) and PC190723-treated (yellow, *n* = 4705 cells) JE2 *S. aureus* cells. **b** Analysis of cell cycle progression in a *S. aureus* strain (control strain BCBMS14 psg-RNA_spy_-2, *n* = 3429 cells) and following CRISPR interference-mediated knockdown of *dnaA* expression (LCML1, *n* = 1647 cells). Cells are classified into three distinct cell cycle phases. Phase 1: spherical cells lacking a septum; Phase 2: slightly elongated cells undergoing septum; Phase 3: cells with a complete, closed septum. All scale bars are 1 µm.

A practical application of quantitative morphological analysis is the detection and characterisation of drug-induced morphological changes. For example, treatment of wild-type JE2 *S. aureus* with PC190723^14^, an FtsZ inhibitor which halts cell division, is known to induce a distinct phenotype: cells become enlarged and are arrested in the first stage of the cell cycle^15^, a stage typically associated with increased roundness. As illustrated in panel a of Fig. 3, mAIcrobe accurately detects and quantifies these morphological changes, underscoring its utility in phenotypic drug screening.

### Classification

A principal strength of mAIcrobe is its adaptable classification system, which is powered by a convolutional neural network (CNN). This system is designed for flexibility and can be fine-tuned to address a variety of biological questions, including cell cycle analysis and antibiotic phenotyping.

The classification module employs a CNN architecture previously developed for cell cycle analysis of *S. aureus*^8^. For example, in images of *S. aureus* cells in which the essential DNA replication initiator protein DnaA^16,17^ was depleted using CRISPR interference (CRISPRi)^18^, mAIcrobe identified altered cell cycle progression (Fig. 3b). This analysis reveals quantifiable differences in cell division timing, which may provide new insights into the role of DnaA in cell cycle regulation.

**Fig. 4:**
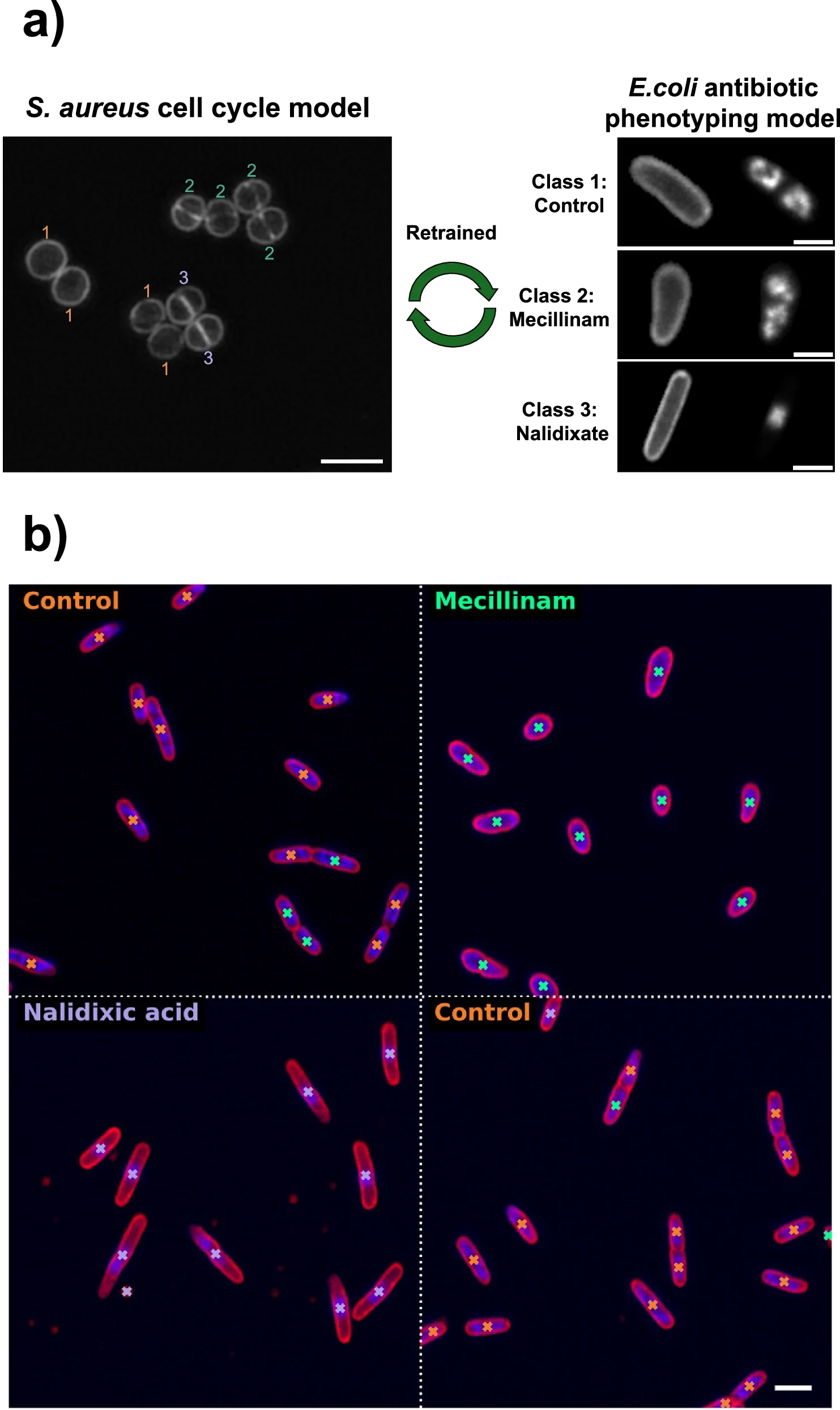
Adaptable classification model in mAIcrobe. **a** SIM image of *S. aureus* labeled with membrane dye Nile Red. Orange, green and purple numbers indicate automatically classified cells in phases 1, 2 or 3, respectively, using mAIcrobe’s pretrained classification model. **b** Image obtained by stitching together multiple fields of view showcasing different drug treatments. mAIcrobe classification model was fine-tuned to classify *E. coli* cells as control or under the effect of different antibiotics (mecillinam and nalidixic acid). Small crosses indicate classification results (orange for control, green for mecillinam and purple for nalidixic acid). Scale bars are 2 µm (*S. aureus* (**a**)) and 3 µm (*E. coli* in (**a**, **b**)).

To support adaptation to diverse experimental conditions and applications beyond cell cycle analysis, a codeless Jupyter notebook (Supplementary Table 1) is provided for straightforward model retraining and fine-tuning. This approach lowers barriers to the development of custom analysis pipelines. The adaptability of the classification system is demonstrated in Fig. 4, which shows the *S. aureus* cell cycle model retrained for antibiotic phenotype detection in *E. coli* (Supplementary Fig. 3)^9^. A video guide for classifier retraining and data annotation within the napari-mAIcrobe plugin is also available (Supplementary Table 2).

### High-content analysis

mAIcrobe also supports high-throughput workflows through a batch analysis mode designed for experiments containing multiple fields of view. In this mode, users organise images into field-of-view subfolders and define filename patterns for the base, membrane and optional DNA channels. The same segmentation algorithms and downstream analysis routines available in the interactive interface can then be applied across the full dataset, including optional channel alignment, septum detection, cell-cycle classification, colocalisation analysis and per-cell morphological measurements. The pipeline writes segmentation masks and labels, per-field reports, a merged analysis table and an error log, enabling large experiments to be processed reproducibly while preserving traceability for each image.

For experiments on dynamic processes, mAIcrobe includes tools for time-lapse analysis and drift correction. Image stacks can be aligned by estimating frame-to-frame drift through cross-correlation and applying the same correction across channels. Once aligned, segmentation and cell measurements can be performed frame by frame to quantify temporal changes in cellular properties. As an example, time-lapse analysis enables extraction of single-cell fluorescence measurements over time, as shown in Supplementary Fig. 4.

## Discussion

mAIcrobe addresses key limitations in current bacterial image analysis workflows by offering a unified framework that integrates deep learning approaches with practical accessibility. Offering a variety of segmentation models constitutes a substantial improvement over single-algorithm methods, as demonstrated by comparative analysis across diverse bacterial morphologies and imaging modalities. Although tools such as eHooke have contributed significantly to the field, they remain constrained by algorithm-specific limitations and biological restrictions, which reduce their broader applicability.

The integration of StarDist, CellPose and custom U-Net models within mAIcrobe enables the selection of optimal segmentation approaches for specific experimental conditions. This flexibility is essential given the morphological diversity among bacterial species and the range of microscopy techniques used in contemporary microbiology. Validation across *S. aureus*, *E. coli*, *S. pneumoniae* and *B. subtilis* demonstrates that the multi-model approach maintains high segmentation accuracy while accommodating diverse cell shapes and imaging protocols.

The adaptable classification system constitutes a key innovation, facilitating the transition from fixed-purpose tools to customisable analysis platforms. By offering accessible retraining protocols through Jupyter notebooks, mAIcrobe reduces technical barriers that have previously limited the adoption of machine learning in bacterial microscopy. The adaptation of the model from *S. aureus* cell cycle classification to *E. coli* antibiotic phenotyping demonstrates the framework’s capacity to address diverse biological questions. Jupyter notebooks, which can be used locally or via Google Colab, enable users to retrain the classification model with minimal computational expertise (Supplementary Table 1).

The morphological measurement capabilities enable comprehensive quantitative profiling of bacterial phenotypes. This functionality is particularly valuable for detecting morphological changes indicative of key biological processes, as demonstrated in analyses of DnaA depletion effects and antibiotic-induced morphological alterations. The ability to export quantitative data in standard formats facilitates integration with statistical analysis workflows and supports reproducible research.

Integration with the napari ecosystem offers strategic advantages for long-term sustainability and community adoption. In contrast to standalone software requiring independent maintenance and feature development, napari plugins benefit from shared infrastructure, advanced visualisation capabilities and an active development community. This approach ensures that mAIcrobe evolves in parallel with advances in the broader image analysis field while maintaining compatibility with complementary tools.

## Conclusions

mAIcrobe offers a comprehensive set of computational tools for bacterial microscopy analysis, delivering a unified solution to the fragmented landscape of existing software (see Supplementary Table 5). The principal innovation of the framework is its seamless integration of multiple segmentation algorithms with adaptable classification models, enabling comprehensive analysis across diverse bacterial species and experimental conditions without requiring transitions between different software packages.

Empirical validation indicates that mAIcrobe’s multi-model approach maintains high analytical performance while substantially expanding the range of addressable biological questions. Demonstrated applications, including the detection of cell cycle defects in DnaA-depleted *S. aureus* and the characterisation of antibiotic-induced morphological changes, highlight the framework’s capacity to reveal biologically relevant phenotypes.

Integration with the napari ecosystem positions mAIcrobe as a forward-looking solution that addresses both current analytical needs and future scalability requirements. By leveraging napari’s extensible architecture and active development community, the framework ensures long-term sustainability and seamless integration with complementary analysis tools. The open-source implementation and accessible retraining protocols broaden access to advanced image analysis capabilities, potentially accelerating discovery across multiple areas of bacterial cell biology.

With the growing demand for sophisticated analytical approaches to address complex biological questions, mAIcrobe provides a robust foundation for next-generation bacterial microscopy analysis. The modular design and extensible architecture enable the incorporation of future methodological advances while maintaining the accessibility and reliability necessary for routine research. This combination of current capability and future adaptability establishes mAIcrobe as a valuable addition to the computational microbiology toolkit.

## Methods

### Image acquisition

The datasets of *S. aureus* strains, JE2^16^, COL^19^, BCBMS14 psg-RNA_spy_-2, LCML1^18^ and *S. pneumoniae* Pen6^20^ (Supplementary Table 6) were acquired in-house.

Overnight cultures of BCBMS14 psg-RNA_spy_-2 and LCML1 were back-diluted 1:500 into 10 mL of tryptic soy broth (TSB, Difco) media containing 10 μg/ml chloramphenicol (Sigma-Aldrich) and grown at 37 °C for 1 h. After 1 h, anhydrotetracycline (aTc, Sigma-Aldrich) was added to the medium at a final concentration of 100 ng/ml. After another hour, a 1 mL aliquot of each culture was incubated with 2.5 μg/mL NileRed (Invitrogen) for 5 min at 37 °C with shaking. The culture was pelleted (10,000 rpm for 1 min), supernatant was removed, and the pellet was resuspended in 30 μL of phosphate-buffered saline (PBS, NaCl 137 mM, KCl 2.7 mM, Na_2_HPO_4_ 10 mM, KH_2_PO_4_ 1.8 mM). One microliter of the resuspended culture was then placed on a thin layer of 1.2% (w/v) agarose (TopVision Thermo Fisher Scientific) in PBS and imaged via structured illumination microscopy (SIM).

An overnight culture of *S. aureus* strain JE2 was back-diluted 1:200 into 10 mL of fresh TSB media and grown at 37 °C until cells reached mid-exponential growth phase (OD600 of 0.8). Afterwards, a 1 mL aliquot of culture was incubated with 5 μg/mL Nile Red (Invitrogen) and 1 μg/mL Hoechst 33342 (Invitrogen) for 5 min at 37 °C with shaking. Culture was then centrifuged, washed with 1 mL of 1:3 (vol/vol) TSB/PBS solution, and resuspended in 20 μL of the same solution. Cells were mounted on microscope slides covered with a layer of 1.2% (w/v) agarose in PBS and imaged via structured illumination microscopy (SIM).

SIM was performed using an Elyra PS.1 microscope (Zeiss) with a Plan-Apochromat 63×/1.4 oil DIC M27 objective. SIM images were acquired using three grid rotations, with a 34-μm grating period for the 561-nm laser (100 mW) and 23 μm grating period for the 405-nm laser (50 mW). Images were captured using a Pco.edge 5.5 camera and reconstructed using ZEN software (black edition, 2012; version 8.1.0.484) on the basis of a structured illumination algorithm, with synthetic, channel-specific optical transfer functions and noise filter settings ranging from 6 to 8.

Overnight cultures of *S. pneumoniae* Pen6 strain were back-diluted 1:50 into 5 mL of fresh C medium supplemented with yeast extract (0.8% Difco Laboratories) (C+Y media). C medium was prepared as described in ref. ^21^. Cells were grown at 37 °C to early exponential phase (OD600 0.2–0.3). A 1 mL aliquot of the culture was centrifuged (10,000 rpm for 1 min), and the pellet was resuspended in 30 μL of pre-C medium^21^. Two microliters of the resuspended culture were then placed on a thin layer of 1.2% (w/v) agarose in pre-C medium^21^ media and imaged using a Zeiss Axio Observer microscope equipped with a Plan-Apochromat 100×/1.4 oil Ph3 objective, a Retiga R1 CCD camera (QImaging), a white-light source HXP 120 V (Zeiss) and the software ZEN blue v2.0.0.0 (Zeiss).

For the timelapse dataset, a culture of strain COL was grown overnight, back-diluted to 1:200 and grown at 37 °C until it reached an OD600 nm of approximately 0.4. To label the cell membrane a 1 mL aliquot of culture was incubated with Nile Red at a final concentration of 10 μg/mL for 5 min at room temperature. The aliquots were then washed with 1 mL PBS, resuspended in 20 μL of PBS and mounted on microscopy slides covered with a thin layer of agarose (1.2% in PBS) and imaged using the Zeiss Axio Observer microscope indicated above. For acquisition of the Nile Red channel, the filter Brightline TXRED-4040B (Semrock USA) was used. Cells were imaged every 5 min for 2 h.

### Biological image datasets

Datasets of *B. subtilis* expressing FtsZ-GFP (strain SH130, PY79 Δ*hag ftsZ::ftsZ-gfp-cam*^22^), which was used to train a U-Net segmentation model, and *E. coli* (strain NO34^23^) exposed to various antibiotics, which was used to train a classification network, are publicly available in^9^ alongside their annotations.

The dataset of *S. pneumoniae* Pen6 that was used to test and train a U-Net segmentation model was acquired in-house and is available on Zenodo (https://doi.org/10.5281/zenodo.17306839).

The *S. aureus* dataset containing untreated and PC190723-treated JE2 cells labeled with Nile Red is publicly available in ref. ^24^. The same dataset, alongside in-house acquired images of BCBMS14 psg-RNA_spy_-2, was used to train and test a StarDist segmentation model and can be found in Zenodo (https://doi.org/10.5281/zenodo.17306839).

The dataset of WT JE2 *S. aureus* cells labeled with Nile Red and Hoechst, used for validating morphometrics, is available in Zenodo (https://doi.org/10.5281/zenodo.17306839).

The *S. aureus* dataset containing the CRISPRi-depleted strain (LCML1) and its respective control (BCBMS14 psg-RNA_spy_-2), used to test the pretrained *S. aureus* cell cycle classification model, was acquired in-house and is available on Zenodo (https://doi.org/10.5281/zenodo.17306839).

The *S. aureus* timelapse dataset of strain COL labeled with NileRed was acquired in-house and is available on Zenodo (https://doi.org/10.5281/zenodo.17306839.

A comprehensive list of all biological datasets used in this study can be found in Supplementary Table 7.

### Segmentation networks

The StarDist model used for *S. aureus* segmentation was trained using a dataset of untreated (10 FoVs) and PC190723- treated (12 FoVs) JE2 *S. aureus* labeled with NileRed. The training dataset is the dataset available in ref. ^24^. The test dataset contains 3 FoVs of BCBMS14 psg-RNA_spy_-2 *S. aureus* strain labeled with Nile Red (Supplementary Table 6)^18^. Both the training and the test dataset are deposited in Zenodo (https://doi.org/10.5281/zenodo.17306839). Training was performed on a Jupyter notebook, adapted from the example notebooks provided by StarDist authors, that can be found in the code repository of this work (Supplementary Table 1).

The U-Net model used for *S. pneumoniae* and *B. subtilis* segmentation was trained on an adapted ZeroCostDL4Mic 2D U-Net notebook^10^, that can be found in the code repository of this work (Supplementary Table 1). The U-Net model was trained to identify background, cell edge and cell interior. To obtain the final label image, scikit-image’s^25^ watershed segmentation was used^26^. First, a mask image is generated by performing the binary union of the cell edge and cell interior. The input to the watershed algorithm is the inverted mask alongside the cell interiors as marker basins. The training and test datasets of *S. pneumoniae* were obtained in-house and are available on Zenodo (https://doi.org/10.5281/zenodo.17306839). The *B. subtilis* training and test datasets are publicly available in ref. ^9^.

For both training datasets, data augmentation was performed using image rotations and flips. The hyperparameters of each model can be found in Supplementary Table 8.

### Classification network

The classification networks trained on *E. coli* data are CNNs with an architecture described in ref. ^8^. These networks were retrained using the *E. coli* antibiotic phenotyping dataset from ref. ^9^. The fields of view pertaining to the control condition plus those corresponding to exposure to mecillinam and nalidixic acid were split into the DNA and membrane channels, and the membrane channel was segmented using the CellPose cyto3 model^12^. Individual cell crops were extracted from the segmented fields of view using mAIcrobe to generate the final dataset needed for training and testing. In short, cell crops were masked to remove background, and their intensity was normalised between 0 and 1. Each crop was then padded to the nearest square and resized to 100 × 100 pixels. In total, the training dataset contained 1164 *E. coli* cell crops while the test dataset contained 414 cells. Five different models were trained, each with increasing levels of data augmentation (no augmentation, 3×, 12×, 24×, 72×) as described in Supplementary Table 9. All networks were trained for 200 epochs with a batch size of 32, a learning rate of 0.0005 and a validation split of 20% (random split). Training was done using a Jupyter notebook^27^ available in our GitHub repository (Supplementary Table 1).

### Image quality deterioration

To artificially deteriorate the quality of the image crop used in Supplementary Fig. 2, an ideal resolution of 100 nm and a Gaussian point spread function (PSF) were assumed. The image was then blurred with a Gaussian kernel with a standard deviation of 2.3 pixels in order to approximately halve the resolution to 200 nm.

The signal-to-noise ratio (SNR) of the original crop was estimated by calculating the mean intensity after subtracting the minimum intensity of the crop, and dividing it by the standard deviation of the background intensity, as determined by a separate crop of the same image with no cells present. To approximately halve the SNR, Gaussian noise with a standard deviation of 3 times the original background standard deviation must be added. To account for the previous blurring step, which flattened the image, we instead added Gaussian noise with a standard deviation of 4 times the original background standard deviation.

## Supplementary information

Supplementary_information

Supplementary_Data1

Supplementary_Data1

## Data Availability

All the segmentation models and the classification model are available via the mAIcrobe GitHub repository (https://github.com/henriqueslab/maicrobe). All the data used in this study is publicly available. The datasets of *B. subtilis* and *E. coli* are available in ref. ^9^. The datasets of *S. aureus* strains, JE2, COL, BCBMS14 psg-RNA_spy_-2 and LCML1, and the dataset of *S. pneumoniae* Pen6 strain were acquired in-house and are available on Zenodo (https://doi.org/10.5281/zenodo.17306839). The numerical source data is also made available alongside the manuscript in Supplementary Data 1, containing one tab per figure panel, named accordingly. A comprehensive list of all models and the respective training and test datasets can be found in Supplementary Table 7.

## Code availability

Source code for the napari-mAIcrobe plugin version 1.0.0 is open source and made available in ref. ^28^. The plugin is also available and installable through PyPI (https://pypi.org/project/napari-mAIcrobe/). Notebooks for training the classification model can be found in the notebooks folder of the mAIcrobe repository. Video tutorials showcasing the functionality of mAIcrobe are available on YouTube (Supplementary Table 2). Documentation, user guides and tutorials are available at https://maicrobe.henriqueslab.org/. Installation instructions can be found at https://maicrobe.henriqueslab.org/user-guide/getting-started/#installation-recommended.

## References

[1] Schindelin, J., Rueden, C. T., Hiner, M. C. & Eliceiri, K. W. The ImageJ ecosystem: an open platform for biomedical image analysis. *Mol. Reprod. Dev.* **82**, 518–529 (2015). 26153368 10.1002/mrd.22489 PMC5428984

[2] Ducret, A., Quardokus, E. M. & Brun, Y. V. MicrobeJ, a tool for high throughput bacterial cell detection and quantitative analysis. *Nat. Microbiol.* **1**, 16077 (2016). 27572972 10.1038/nmicrobiol.2016.77 PMC5010025

[3] Paintdakhi, A. et al. Oufti: an integrated software package for high-accuracy, high-throughput quantitative microscopy analysis. *Mol. Microbiol.* **99**, 767–777 (2016). 26538279 10.1111/mmi.13264 PMC4752901

[4] Lo, T. W., Cutler, K. J., Choi, H. J. & Wiggins, P. A. OmniSegger: a time-lapse image analysis pipeline for bacterial cells. *PLoS Comput. Biol.* **21**, e1013088 (2025). 40435362 10.1371/journal.pcbi.1013088 PMC12140430

[5] Stirling, D. R. et al. CellProfiler 4: improvements in speed, utility and usability. *BMC Bioinform.* **22**, 433 (2021). 10.1186/s12859-021-04344-9 PMC8431850 34507520

[6] Coppola, S. & Holden, S. micromorph: a Python toolkit for measurement of microbial morphology. Preprint at 10.64898/2025.12.05.692496 (2026).

[7] Sofroniew, N. et al. napari: a multi-dimensional image viewer for Python. 10.5281/zenodo.7276432 (2022).

[8] Saraiva, B. M., Krippahl, L., Filipe, S. R., Henriques, R. & Pinho, M. G. eHooke: a tool for automated image analysis of spherical bacteria based on cell cycle progression. *Biol. Imaging* **1**, e3 (2021). 35036921 10.1017/S2633903X21000027 PMC8724265

[9] Spahn, C. et al. DeepBacs for multi-task bacterial image analysis using open-source deep learning approaches. *Commun. Biol.* **5**, 688 (2022). 35810255 10.1038/s42003-022-03634-z PMC9271087

[10] von Chamier, L. et al. Democratising deep learning for microscopy with ZeroCostDL4Mic. *Nat. Commun.* **12**, 2276 (2021). 33859193 10.1038/s41467-021-22518-0 PMC8050272

[11] Schmidt, U., Weigert, M., Broaddus, C. & Myers, G. Cell detection with star-convex polygons. In *Proc. Medical Image Computing and Computer Assisted Intervention - MICCAI 2018**,* 265–273, 10.1007/978-3-030-00934-2_30 (2018).

[12] Stringer, C., Wang, T., Michaelos, M. & Pachitariu, M. Cellpose: a generalist algorithm for cellular segmentation. *Nat. Methods* **18**, 100–106 (2021). 33318659 10.1038/s41592-020-01018-x

[13] Ronneberger, O., Fischer, P. & Brox, T. U-Net: convolutional networks for biomedical image segmentation. In *Proc. Medical Image Computing and Computer Assisted Intervention - MICCAI 2015*, 234–241, 10.1007/978-3-319-24574-4_28 (2015).

[14] Haydon, D. J. et al. An inhibitor of FtsZ with potent and selective anti-staphylococcal activity. *Science* **321**, 1673–1675 (2008). 18801997 10.1126/science.1159961

[15] Monteiro, J. M. et al. Peptidoglycan synthesis drives an FtsZ-treadmilling-independent step of cytokinesis. *Nature* **554**, 528–532 (2018). 29443967 10.1038/nature25506 PMC5823765

[16] Fey, P. D. et al. A genetic resource for rapid and comprehensive phenotype screening of nonessential *Staphylococcus aureus* genes. *mBio* **4**, e00537-12. 10.1128/mbio.00537-12 (2013). 23404398 PMC3573662

[17] Chaudhuri, R. R. et al. Comprehensive identification of essential *Staphylococcus aureus* genes using Transposon-Mediated Differential Hybridisation (TMDH). *BMC Genom.* **10**, 291 (2009). 10.1186/1471-2164-10-291 PMC2721850 19570206

[18] Reed, P. et al. A CRISPRi-based genetic resource to study essential *Staphylococcus aureus* genes. *mBio* **15**, e0277323 (2024). 38054745 10.1128/mbio.02773-23 PMC10870820

[19] Gill, S. R. et al. Insights on evolution of virulence and resistance from the complete genome analysis of an early methicillin-resistant *Staphylococcus aureus* strain and a biofilm-producing methicillin-resistant *Staphylococcus epidermidis* strain. *J. Bacteriol.* **187**, 2426–2438, 10.1128/jb.187.7.2426-2438.2005 (2005). 15774886 PMC1065214

[20] Zighelboim, S. & Tomasz, A. Penicillin-binding proteins of multiply antibiotic-resistant South African strains of *Streptococcus pneumoniae*. *Antimicrob. Agents Chemother.* **17**, 434–442 (1980). 6903436 10.1128/aac.17.3.434 PMC283805

[21] Tomasz, A. Choline in the cell wall of a bacterium: novel type of polymer-linked choline in pneumococcus. *Science* **157**, 694–697 (1967). 4381896 10.1126/science.157.3789.694

[22] Whitley, K. D. et al. FtsZ treadmilling is essential for Z-ring condensation and septal constriction initiation in *Bacillus subtilis* cell division. *Nat. Commun.* **12**, 2448 (2021). 33907196 10.1038/s41467-021-22526-0 PMC8079713

[23] Ouzounov, N. et al. MreB orientation correlates with cell diameter in *Escherichia coli*. *Biophys. J.* **111**, 1035–1043 (2016). 27602731 10.1016/j.bpj.2016.07.017 PMC5018124

[24] Ferreira, M. G. et al. ReScale4DL: balancing pixel and contextual information for enhanced bioimage segmentation. Preprint at 10.1101/2025.04.09.647871 (2025).

[25] Walt, S. et al. scikit-image: image processing in Python. *PeerJ* **2**, e453 (2014). 10.7717/peerj.453 PMC4081273 25024921

[26] Roerdink, J. B. T. M. & Meijster, A. The watershed transform: definitions, algorithms and parallelization strategies. *Fundam. Inform.* **41**, 187–228 (2000).

[27] Kluyver, T. et al. Jupyter Notebooks—a publishing format for reproducible computational workflows. in *Positioning and Power in Academic Publishing: Players, Agents and Agendas,* 87–90, 10.3233/978-1-61499-649-1-87 (IOS Press, 2016).

[28] Brito, A. D., Saraiva, B. M. & Henriques, R. mAIcrobe: an open-source framework for high-throughput bacterial image analysis, *HenriquesLab/mAIcrobe*. 10.5281/zenodo.21738445 (2026).

