# Supplementary material for: mAIcrobe: an open-source framework for high-throughput bacterial image analysis
Source: Commun AI Comput. 2026 Sep 15;1(1):16. doi: 10.1038/s44488-026-00022-y (PMC13577909; doi:10.1038/s44488-026-00022-y)
Supplement: Supplementary file 1 [file 44488_2026_22_MOESM1_ESM.pdf]

Supplementary Information

mAIcrobe: an open-source framework for high-throughput bacterial image analysis

Supplementary Tables

Notebook	Link
StarDist2D training notebook	https://github.com/HenriquesLab/mAIcrobe/blob/main/notebooks/StarDistSegmentationTraining.ipynb
U-Net2D training notebook	https://github.com/HenriquesLab/mAIcrobe/blob/main/notebooks/U_Net_2D_Multilabel_ZeroCostDL4Mic_adapted.ipynb
CNN classification network training	https://github.com/HenriquesLab/mAIcrobe/blob/main/notebooks/napari_mAIcrobe_cellcyclemodel.ipynb

Sup. Table S1. Jupyter notebooks available as part of mAIcrobe

Description	Link
mAIcrobe – Showcase and basic workflow	https://youtu.be/UQ-dgATCgkU
mAIcrobe – Example full workflow	https://youtu.be/szYAi0zSKSA
mAIcrobe – How to retrain the classifier	https://youtu.be/fO3XxTM3xk8

Sup. Table S2. Videos showcasing mAIcrobe functionality.

Description	Species	Microscopy modality	Reference
S. aureus datasets	*S. aureus*	SIM and Widefield (phase contrast and fluorescence)	This study and [1]
S. pneumoniae U-Net datasets	*S. pneumoniae*	Widefield (phase contrast)	This study
B. subtilis U-Net dataset	*B. subtilis*	Widefield (fluorescence)	[2]
E. coli classification model dataset	*E. coli*	Widefield (fluorescence)	[2]

Sup. Table S3. Experimental conditions for datasets used in this study.

Model	Average IoU	Recall	Precision
StarDist *S. aureus*	0.872	0.999	0.994
U-Net *S. pneumoniae*	0.898	0.998	0.988
U-Net *B. subtilis*	0.840	0.923	1.00

Sup. Table S4. Performance metrics for the segmentation networks used in this study. Average Intersection over Union (IoU), Recall and Precision values were calculated on the respective test datasets. All tests datasets had three FoV's.

Tool	Bacteria focused	Multimethod segmentation	DL segmentation	DL custom training	DL classification	Fluorescence /subcellular analysis	Python/ napari API
mAIcrobe (this work)	✓	✓	✓	✓	✓	✓	✓
MicrobeJ[3]	✓	✗	✗	✗	✗	✓	✗
OmniSegger[4]	✓	✗	✓	✗	✗	✓	✗
Oufti[5]	✓	✗	✗	✗	✗	✓	✗
CellProfiler[6]	✗	✓	✓	✓	✓	✓	✗
micromorph[7]	✓	✗	✓	✗	✗	✗	✓
eHooke[8]	✓	✗	✗	✗	✓	✓	✗

Sup. Table S5. Comparison of differentiating features across bacterial image analysis tools

Name	Description	Reference
	Staphylococcus aureus	
JE2	Derivative of community acquired MRSA	9
COL	Hospital-acquired MRSA	10
BCBMS14 psg-RNA$_{Spy}$-2	JE2 Δ*spa*:$P_{xyl/tet03}$–*dcas9*$_{Spy}$ containing psg-RNA$_{Spy}$-2	11
LCML1	JE2 Δ*spa*:$P_{xyl/tet03}$–*dcas9*$_{Spy}$ containing psg-001_dnaA	11
	Streptococcus pneumoniae	
Pen6	Pen^R, unencapsulated laboratory strain; carries mosaic *pbp* alleles	12

Sup. Table S6. Strains used in this study.

Name	Description	Link
JE2 WT with and without PC190723	Wild-type strain of *S. aureus* with and without PC190723 treatment	https://zenodo.org/records/15169018
Timelapse COL WT	Timelapse of *S. aureus* COL cells labeled with NileRed	https://doi.org/10.5281/zenodo.17306839
psg-RNA$_{spy}$-2 and LCML1	*S.aureus dnaA* CRISPRi knockdown strain and its respective control	https://doi.org/10.5281/zenodo.17306839
StarDist *S. aureus* dataset	Training and test dataset for *S. aureus* StarDist segmentation model	https://doi.org/10.5281/zenodo.17306839
U-Net *S. pneumoniae* dataset	Training and test dataset for *S. pneumoniae* U-Net segmentation model	https://doi.org/10.5281/zenodo.17306839
U-Net *B. subtilis* dataset	Training and test dataset for *B. subtilis* U-Net segmentation model	https://zenodo.org/records/5550968
E. coli classification model dataset	Training and test dataset for mAIcrobe's *E. coli* classification model	https://zenodo.org/records/5551057

Sup. Table S7. Datasets used in this study and their respective repository links

Organism	Microscopy	Network	Train/test images	Epochs	Steps	Image size	Patch size	Batch size	Learning rate	%Validation (random split)	Augmentation	Misc
S. aureus	SIM	StarDist	20/3	400	100	2430x2430	256x256	4	0.0003	15	Random flip and intensity changes	grid 2, 32 rays
S. pneumoniae	Phase contrast	U-Net	16/3	100	70	1392x1040	256x256	4	0.0003	10	VHFlip and 180ºrot	Finetuned from a *S. aureus* U-Net model
B. subtilis	Fluorescence	U-Net	7/3	100	6	1024x1024	512x512	4	0.0005	10	VHFlip and 180ºrot	Finetuned from a *S. aureus* U-Net model

Sup. Table S8. Hyperparameters for the segmentation networks used in this study.

Model	LRUD Flips	Rotations	Total dataset size	Accuracy (%)
NO_AUG	No	No	1164 cells (1x)	73.9%
LRUD	Yes	No	3492 cells (3x)	82.4%
LRUD_90	Yes	Every 90º	13968 cells (12x)	90.1%
LRUD_45	Yes	Every 45º	27935 cells (24x)	89.4%
LRUD_15	Yes	Every 15º	83808 cells (72x)	91.3%

Sup. Table S9. Different antibiotic phenotyping classification models trained. All models were trained using the same hyperparameters as described in the Methods section, only differing on the amount of data augmentation. All models were tested on the same dataset of 414 cells.

Supplementary Figures

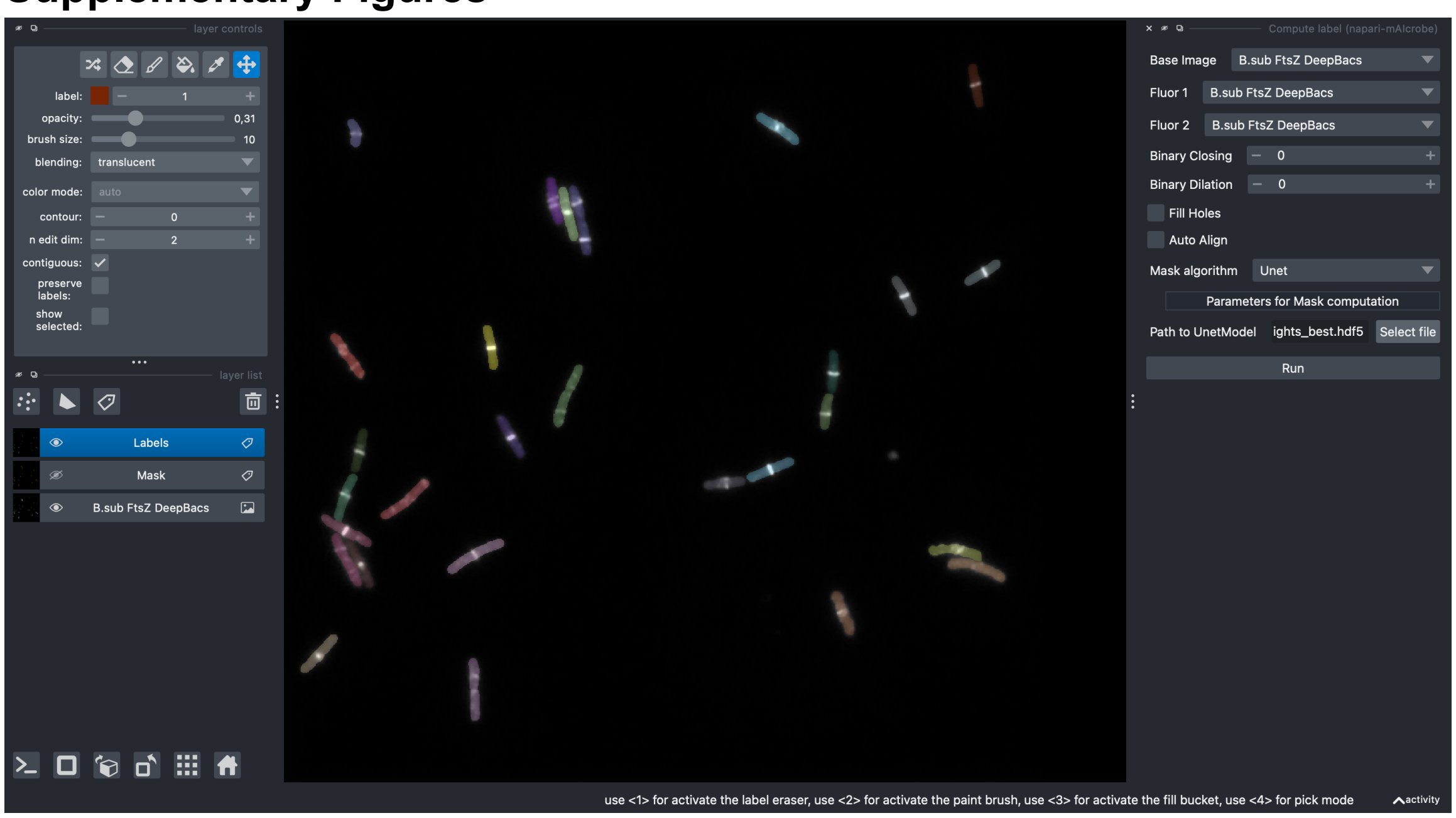

Supp. Fig. S1. Example screenshot of the mAIcrobe napari plugin. In this screenshot, we showcase an image of *B.subtilis* cells being segmented using a U-Net model loaded into the mAIcrobe plugin. The plugin's segmentation interface is visible on the right side of the image, displaying various options and settings for segmentation, that dynamically change according to the segmentation model chosen.

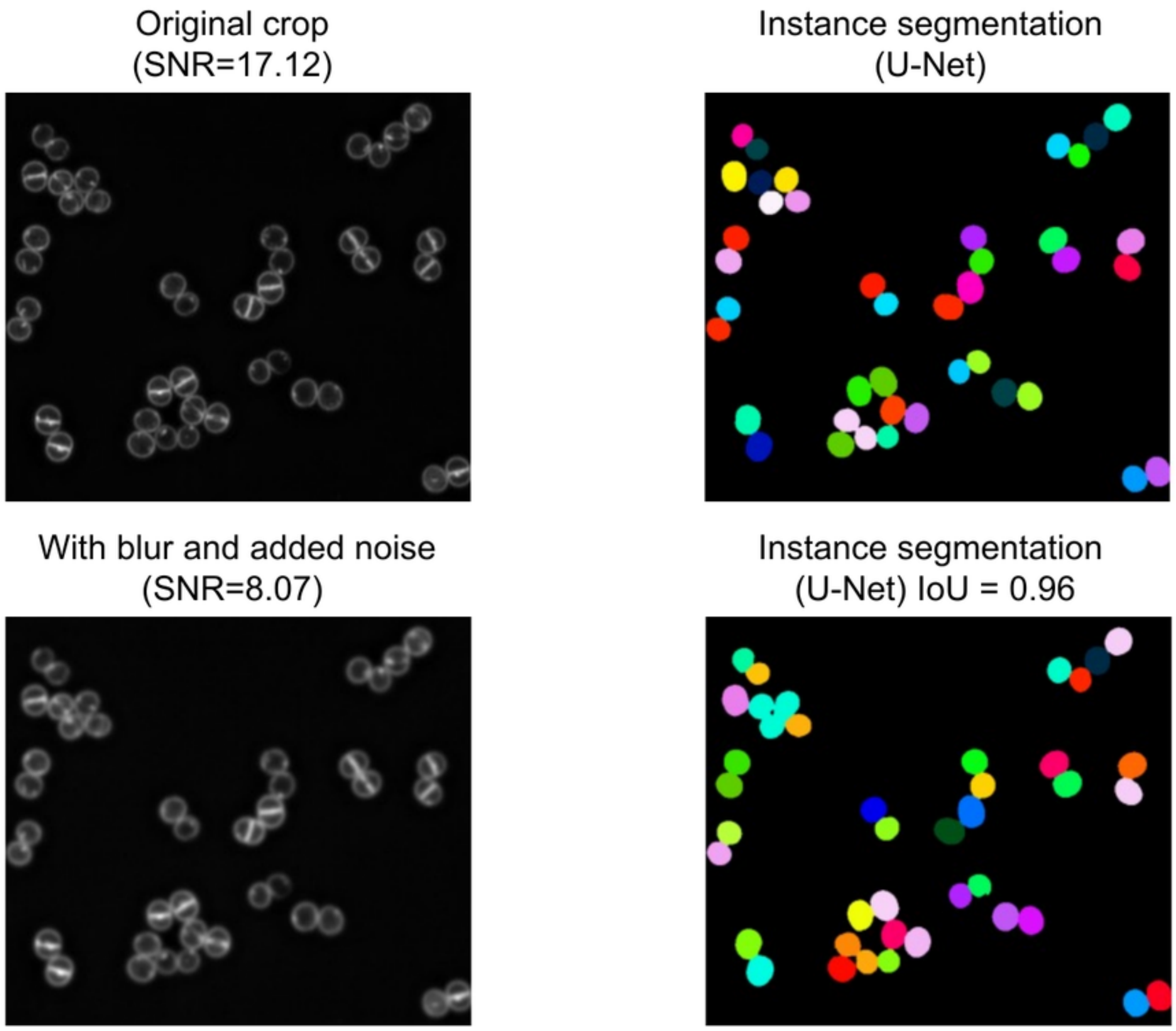

Supp. Fig. S2. SNR and resolution deterioration do not impair accurate segmentation. Top row shows crops of a representative field of view of *S. aureus* JE2 cells imaged with SIM (left) and its segmentation using a mAIcrobe's U-Net pretrained model (right). In the bottom row, the same field of view is shown with added Gaussian noise and a 2D Gaussian blur to simulate approximately half SNR and resolution. The segmentation of the noisy image is still accurate (IoU=0.96 when compared to the original segmentation).

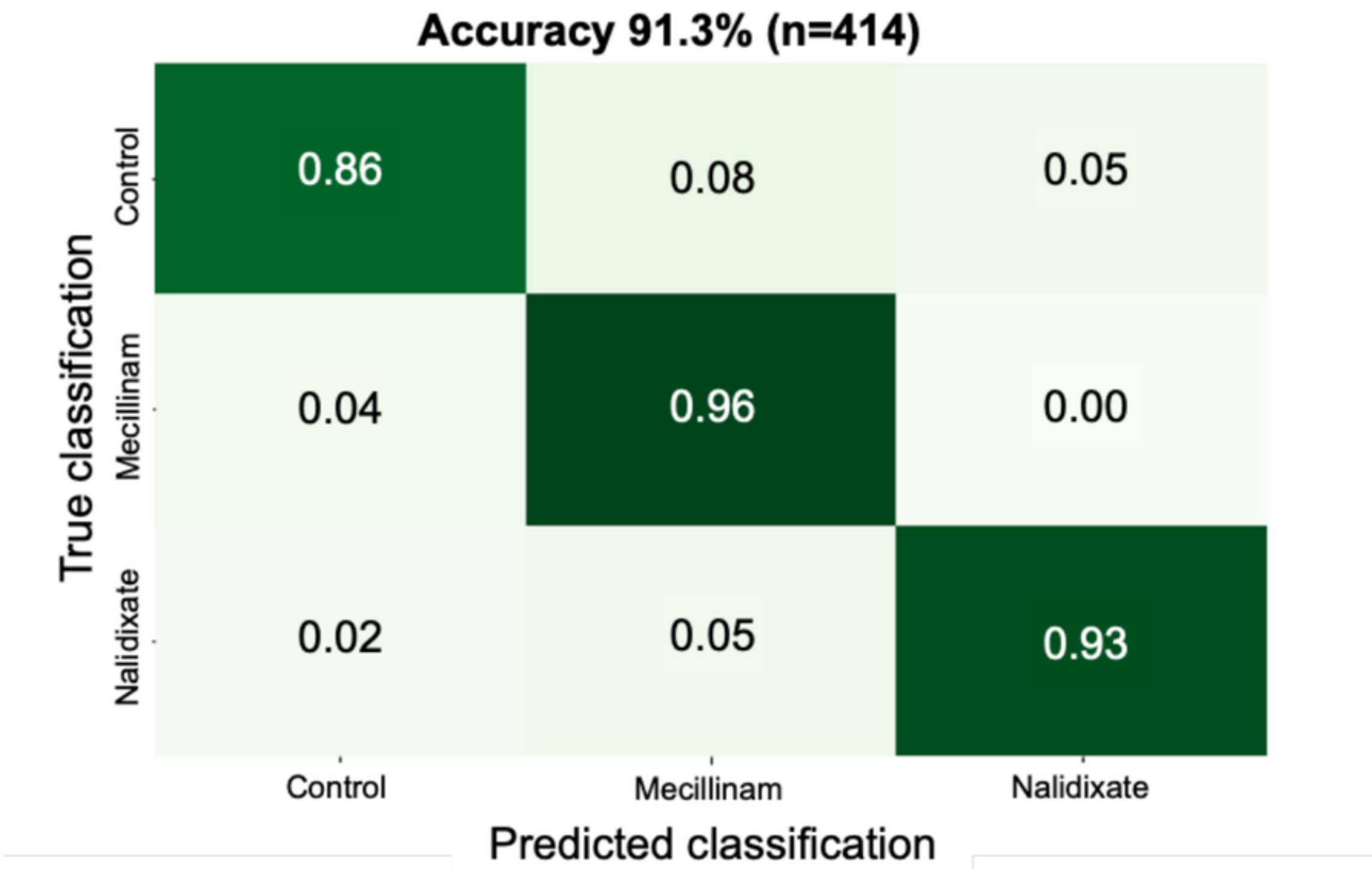

Supp. Fig. S3. Confusion matrix for the LRUD_15 classification model. The confusion matrix shows the performance of the best retrained mAIcrobe CNN on a test dataset of 414 *E. coli* cells.

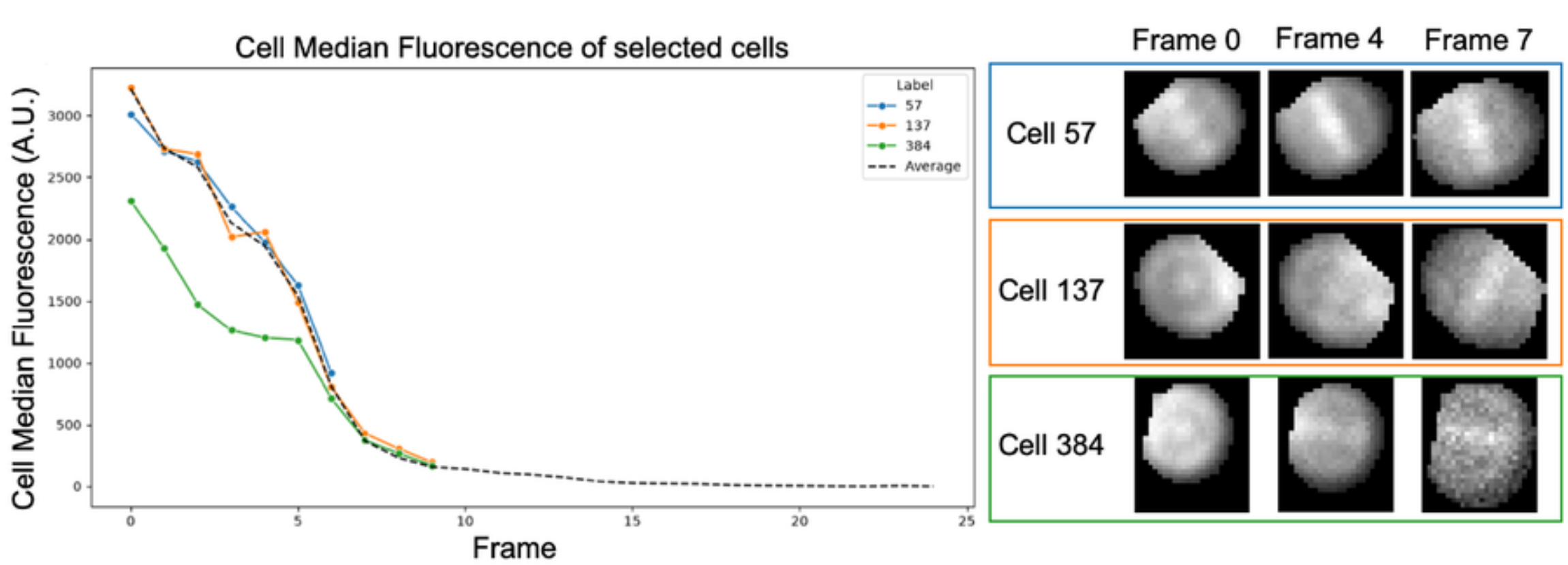

Supp. Fig. S4. Cell median fluorescence measured over time. mAIcrobe analysis of a timelapse allows for quantification of cell metrics over time. Left: median fluorescence of cells labeled with NileRed in a timelapse of *S. aureus* COL cells. The median fluorescence was calculated for each frame of the timelapse and plotted over time. The black dotted line represents the average of the field of view while the colored lines are representative individual cells. Right: small crops of the representative cells highlighted in the left panel.

References

1. Ferreira, M. G. *et al.* ReScale4DL: Balancing Pixel and Contextual Information for Enhanced Bioimage Segmentation. (2025) doi:10.1101/2025.04.09.647871.

2. Spahn, C. *et al.* DeepBacs for multi-task bacterial image analysis using open-source deep learning approaches. *Commun Biol* **5**, 688 (2022).

3. Ducret, A., Quardokus, E. M. & Brun, Y. V. MicrobeJ, a tool for high throughput bacterial cell detection and quantitative analysis. *Nat Microbiol* **1**, 16077 (2016).

4. Lo, T. W., Cutler, K. J., Choi, H. J. & Wiggins, P. A. OmniSegger: A time-lapse image analysis pipeline for bacterial cells. *PLOS Comput. Biol.* **21**, e1013088 (2025).

5. Paintdakhi, A. *et al.* Oufti: an integrated software package for high-accuracy, high-throughput quantitative microscopy analysis. *Mol Microbiol* **99**, 767–777 (2016).

6. Stirling, D. R. *et al.* CellProfiler 4: improvements in speed, utility and usability. *BMC Bioinformatics* **22**, 433 (2021).

7. Coppola, S. & Holden, S. micromorph: a Python toolkit for measurement of microbial morphology. 2025.12.05.692496 Preprint at https://doi.org/10.64898/2025.12.05.692496 (2026).

8. Saraiva, B. M., Krippahl, L., Filipe, S. R., Henriques, R. & Pinho, M. G. eHooke: A tool for automated image analysis of spherical bacteria based on cell cycle progression. *Biol. Imaging* **1**, e3 (2021).

9. Fey, P. D. *et al.* A Genetic Resource for Rapid and Comprehensive Phenotype Screening of Nonessential *Staphylococcus aureus* Genes. *mBio* **4**, 10.1128/mbio.00537-12 (2013).

10. Gill S. R. *et al*. Insights on Evolution of Virulence and Resistance from the Complete Genome Analysis of an Early Methicillin-Resistant *Staphylococcus aureus* Strain and a Biofilm-Producing Methicillin-Resistant *Staphylococcus epidermidis* Strain. *J.Bacteriol* **187**, https://doi.org/10.1128/jb.187.7.2426-2438.2005 (2005)

11. Reed, P. *et al.* A CRISPRi-based genetic resource to study essential *Staphylococcus aureus* genes. *mBio* **15**, e0277323 (2024).

12. Zighelboim, S. & Tomasz, A. Penicillin-binding proteins of multiply antibiotic-resistant South African strains of *Streptococcus pneumoniae*. *Antimicrob Agents Chemother* **17**, 434–442 (1980).
