# Supplementary material for: mAIcrobe: an open-source framework for high-throughput bacterial image analysis
Source: Commun AI Comput. 2026 Sep 15;1(1):16. doi: 10.1038/s44488-026-00022-y (PMC13577909; doi:10.1038/s44488-026-00022-y)
Supplement: Supplementary file 2 [file 44488_2026_22_MOESM2_ESM.docx]

# Supplementary Data 1

Source data for the mAIcrobe manuscript. The workbook holds the numerical source data underlying Fig. 3, Supplementary Fig. 3, Supplementary Fig. 4 and Supplementary Table 9, with one tab per figure panel, named accordingly.

**Fig3a_WT.** Per cell area and eccentricity of JE2 WT *S. aureus*.

**Fig3a_PC.** Per cell area and eccentricity of JE2 WT *S. aureus* treated with PC190723.

**Fig3b_BCBMS14 psg-RNAspy-2.** Cell cycle phases of single cells of the BCBMS14 psg-RNAspy-2 strain of *S. aureus*.

**Fig3b_LCML1.** Cell cycle phases of single cells of the LCML1 strain of *S. aureus*.

**SFig3_STable9.** Classification results of four *E. coli* antibiotic phenotyping models over the same test dataset.

**SFig4.** Average cell median fluorescence of COL WT *S. aureus* cells imaged over time, and the cell median fluorescence over time of three representative cells.

A Caption tab in the workbook repeats these descriptions.
